# Deep Vein Thrombosis as a Rare Post-procedural Complication After Uterine Artery Embolization: A Case Report

**DOI:** 10.7759/cureus.39716

**Published:** 2023-05-30

**Authors:** Swati M Dahiphale, Jyotsana Potdar, Apoorva Dave, Neema Acharya, Sharmeen I Memon

**Affiliations:** 1 Obstetrics and Gynaecology, Jawaharlal Nehru Medical College, Datta Meghe Institute of Higher Education & Research, Wardha, IND

**Keywords:** interventional radiology-guided embolization, gynecology, dvt, aub, uae, uterine artery embolization (uae), deep vein thrombosis (dvt), abnormal uterine bleeding (aub)

## Abstract

When a patient is undergoing uterine artery embolization (UAE) which is considered a modality that is safer than surgical management for abnormal uterine bleeding (AUB), one must acknowledge as a surgeon the existence of rare but serious complications such as deep vein thrombosis (DVT). We encountered such a case where a 34-year-old female (para-3 living-3) with AUB and severe anemia because of heavy bleeding required multiple blood transfusions and was treated with UAE. The procedure was uneventful and the patient was discharged. However, later she presented with DVT of the right lower limb which was promptly managed with an inferior vena cava filter implant and thrombolysis, which prevented life-threatening sequelae such as pulmonary embolism and, potentially, death. Therefore, one must be vigilant about such complications despite UAE being a safer alternative to surgical management for gynecological complaints.

## Introduction

In India, the reported prevalence of abnormal uterine bleeding (AUB) is 17-18% in women of reproductive age [1]. Treatment is based on the cause according to the PALM-COEIN. It is a system approved and adopted by the International Federation of Gynecology and Obstetrics (FIGO) where PALM are structural causes of AUB (P: polyps, A: adenomyosis, L: leiomyomas/fibroids, M: malignancy and hyperplasia) and COEIN denotes non-structural and systemic causes of AUB (C: coagulopathies, O: ovulatory dysfunction, E: endometrial pathologies, I: iatrogenic, N: not yet classified) [2].

Medical management is still the most widely used modality for treating AUB [3]. However, many patients do not respond and get no relief despite long-term medical management. These patients require surgical interventions, such as hysteroscopy with dilatation and curettage, polypectomy, myomectomy, or endometrial ablation, which are beneficial for structural abnormalities of the uterus leading to AUB [4]. For women who do not respond to medical therapy or when surgical intervention is not satisfactory, a hysterectomy is the definitive management for AUB, performed only when the woman does not desire future pregnancy. Uterine artery embolization (UAE) is an emerging modality for effectively treating AUB in reproductive and postmenopausal women [5]. It offers early ambulation, fewer hospital stays, and better cosmetic appeal. Unilateral UAE also provides satisfactory symptomatic relief in many patients [6].

Additionally, preoperative UAE reduces blood loss during subsequent definitive surgical management. Although it is generally regarded as a safe procedure, death due to pulmonary embolism after UAE has been reported [7]. Hence, physicians should be aware of the existence of lethal complications such as deep vein thrombosis (DVT) and pulmonary embolism that may occur in a patient undergoing UAE. Here, we present a case where the patient developed DVT post-UAE which was quickly and aptly managed through admission.

## Case presentation

A 34-year-old woman (para 3, living 3) presented to the gynecology outpatient department with complaints of heavy menstrual bleeding for 2 weeks every month for the past three years for which medical therapy was given in the form of OC pills and tranexamic acid. However, the patient did not get relief and heavy menstrual bleeding persisted. On general examination, the patient had moderate pallor with pale conjunctiva and skin. The systemic examination yielded results within the normal range, revealing normal blood pressure and cardiovascular functions. A 12-week gravid uterus was discovered during a gynecological examination, with ultrasonography revealing a bulky uterus with an intramural fibroid of 48 mm × 52 mm in the anterior wall of the uterus. Her hemoglobin level was 8.2 g/dL. Two units of packed cells were transfused to correct anemia, and the post-transfusion hemoglobin was 10.4 g/dL.

**Figure 1:**
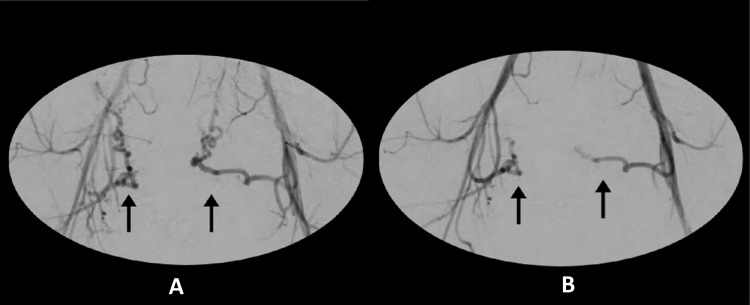
Digital subtraction angiography images of the uterine artery during the uterine artery embolization procedure. (A) Dilated and tortuous uterine arteries before embolization. (B) Effective embolization of the uterine arteries.

The patient desired conservative management despite not wanting to conceive in the future. Taking her preference and general condition into consideration, surgical modalities were excluded and UAE was considered to be an appropriate therapeutic modality. The right femoral artery was accessed using a 5F sheath. Bilateral iliac arteries were then cannulated using a 5F cobra catheter followed by selective cannulation of bilateral uterine arteries. Embolization was done using 300-500 µ polyvinyl alcohol particles (Figures 1A, 1B).

**Figure 2:**
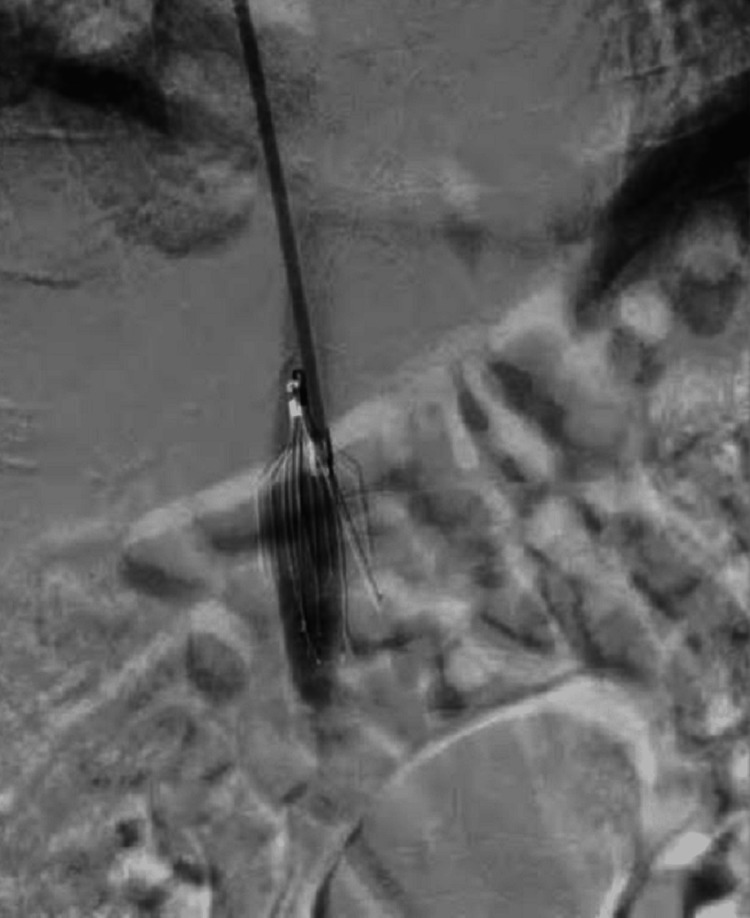
Digital subtraction angiography image showing Denali filter in the inferior vena cava.

The procedure was uneventful and the patient was discharged three days later. However, two weeks later, she presented to the emergency department with a complaint of a swollen painful right lower limb. She developed DVT after UAE. The patient was admitted and managed with an inferior vena cava (IVC) filter. Denali IVC filter was implanted in the IVC through a right internal jugular vein access (Figure 2). Right popliteal vein access was taken, and with mechanical maceration and aspiration, a thrombus was aspirated. The patient was advised prophylactic anticoagulant therapy with heparin 20 mg once daily for three months. Six months after UAE, the patient had regular menses with complete relief of her symptoms.

## Discussion

The safety of UAE for uterine fibroids has been confirmed by large-scale trials [8]. Some common side effects of UAE are post-embolization syndrome seen in 20-30% of cases, which includes fever, pain, nausea, and raised markers of inflammation [9]. It is a typical side effect of a solid organ. Commonly reported consequences of UAE are prolonged or poorly controlled vague pain, infections of the genitourinary tract leading to endometritis, urinary tract infection leading to urinary retention, pyomyoma, delayed return of menses, prolonged vaginal discharge, urinary retention, and injury to the neurovascular bundle during the procedure at the access site. Some uncommon complications seen are the expulsion of submucosal and cervical fibroid (8%) [10], premature ovarian failure (1-2%), septicemia, infection needing rescue hysterectomy (<1%), and amenorrhea. Rare major complications post-UAE include unintended embolization of a leiomyosarcoma, uterine necrosis, acute renal failure, sepsis, and death secondary to DVT or pulmonary embolism. As UAE is becoming accessible to peripheral areas, more people are seeking out this modality for the management of AUB. At the same time, physicians must bear in mind the inadvertent complications that may turn fatal if neglected. Patients must be educated about possible adverse effects and advised to follow up. These complications are uncommon but are of significant importance and need to be diagnosed early and managed promptly.

## Conclusions

UAE is a safe, effective, and well-tolerated alternative to surgery in women with AUB which might be due to multiple causes ranging from polyp to dysfunctional uterine bleeding to adenomyosis. Most complications are minor and easily manageable which makes UAE a desired alternative to surgery. However, in some cases, UAE may fail and patients have to undergo surgery to achieve the therapeutic effect. However, in all cases, physicians should be aware of catastrophic serious complications of this simple procedure such as DVT and pulmonary embolism which may turn fatal. Hence, regular follow-up, which is not commonly seen after UAE, should be advised to prevent and diagnose critical complications at the earliest and as seen in this case.
